# Noninvasive Monitoring of Allogeneic Stem Cell Delivery with Dual-Modality Imaging-Visible Microcapsules in a Rabbit Model of Peripheral Arterial Disease

**DOI:** 10.1155/2019/9732319

**Published:** 2019-03-14

**Authors:** Yingli Fu, Clifford R. Weiss, Dorota A. Kedziorek, Yibin Xie, Ellen Tully, Steven M. Shea, Meiyappan Solaiyappan, Tina Ehtiati, Kathleen Gabrielson, Frank H. Wacker, Jeff W. M. Bulte, Dara L. Kraitchman

**Affiliations:** ^1^Russell H. Morgan Department of Radiology and Radiological Science, Johns Hopkins University, Baltimore, MD, USA; ^2^Cellular Imaging Section and Vascular Biology Program, Institute for Cell Engineering, Johns Hopkins University School of Medicine, Baltimore, MD, USA; ^3^Siemens Corporation, Corporate Technology, Baltimore, MD, USA; ^4^Department of Molecular & Comparative Pathobiology, Johns Hopkins University, Baltimore, MD, USA; ^5^Department of Radiology, Hannover Medical School, Hannover, Germany

## Abstract

Stem cell therapies, although promising for treating peripheral arterial disease (PAD), often suffer from low engraftment rates and the inability to confirm the delivery success and track cell distribution and engraftment. Stem cell microencapsulation combined with imaging contrast agents may provide a means to simultaneously enhance cell survival and enable cell tracking with noninvasive imaging. Here, we have evaluated a novel MRI- and X-ray-visible microcapsule formulation for allogeneic mesenchymal stem cell (MSC) delivery and tracking in a large animal model. Bone marrow-derived MSCs from male New Zealand White rabbits were encapsulated using a modified cell encapsulation method to incorporate a dual-modality imaging contrast agent, perfluorooctyl bromide (PFOB). PFOB microcapsules (PFOBCaps) were then transplanted into the medial thigh of normal or PAD female rabbits. *In vitro* MSC viability remained high (79 ± 5% at 4 weeks of postencapsulation), and as few as two and ten PFOBCaps could be detected in phantoms using clinical C-arm CT and ^19^F MRI, respectively. Successful injections of PFOBCaps in the medial thigh of normal (*n* = 15) and PAD (*n* = 16) rabbits were demonstrated on C-arm CT at 1-14 days of postinjection. Using ^19^F MRI, transplanted PFOBCaps were clearly identified as “hot spots” and showed one-to-one correspondence to the radiopacities on C-arm CT. Concordance of ^19^F MRI and C-arm CT locations of PFOBCaps with postmortem locations was high (95%). Immunohistological analysis revealed high MSC survival in PFOBCaps (>56%) two weeks after transplantation while naked MSCs were no longer viable beyond three days after delivery. These findings demonstrate that PFOBCaps could maintain cell viability even in the ischemic tissue and provide a means to monitor cell delivery and track engraftment using clinical noninvasive imaging systems.

## 1. Introduction

Peripheral arterial disease (PAD) affects approximately 8-12 million Americans and its prevalence increases exponentially with age [1]. Patients with PAD are often at high risk for cardiovascular and cerebrovascular morbidity and mortality [2]. However, current revascularization treatments for PAD, such as surgical bypass and angioplasty, have significant complications and approximately one-third of PAD patients are not amenable to these therapies due to the extent and distribution pattern of their atherosclerosis [3]. Thus, alternative treatment strategies for revascularization of ischemic limbs are critically needed. Recent clinical and preclinical studies using autologous stem cells have shown promising results in enhancing neovascularization and tissue perfusion in PAD patients [4–6]. Nevertheless, individuals with PAD often lack the endogenous arteriogenic/angiogenic responses due to dysfunction of their native stem cells [7, 8]. Therefore, allogeneic or xenogeneic stem cell-based therapeutic approaches might be more realistic to provide a ready-to-use, off-the-shelf, cellular product for PAD patients.

However, current stem cell therapies suffer from low engraftment, primarily due to the early immunorejection and destruction of transplanted cells shortly after administration [9–11] as well as lack of the ability to noninvasively monitor the delivery, distribution, and engraftment of transplanted cells longitudinally. Therefore, the therapeutic efficacy of cellular therapies could be improved if methods to protect transplanted cells from host immunorejection and to enable *in vivo* imaging visualization were available. Cell microencapsulation with appropriate matrices theoretically can immunoprotect the cells by blocking the passage of antibodies and other immune mediators (e.g., complement and T cells) while allowing the free exchange of oxygen, nutrient, and therapeutic proteins between the encapsulated cells and their surroundings [12]. Since the introduction of this concept, various cell types, including pancreatic islets [13, 14] and stem cells [15], have been encapsulated to explore its immunoisolation potential for the cells in various disease settings. However, clinical translation has been hampered by the low graft survival, which, in part, may be attributed to fibrotic outgrowth of the microcapsules [16, 17] or hypoxia-induced necrotic cell death [18].

We investigated here whether impregnating the microcapsules with perfluorooctyl bromide (PFOB) could maintain MSC viability and enable cell monitoring with clinical MRI and X-ray *in vivo*. PFOB is a multifunctional perfluorocarbon that can be used as a contrast agent for MRI, CT, and ultrasound imaging, including cell labeling [19]. Since there is negligible endogenous fluorine in the body, perfluorocarbon-impregnated microcapsules will enable “hot spots” ^19^F MRI. Recently, perfluorocarbons have been used in first in-human clinical trial for labeling stromal vascular fraction therapeutic cells [20]. Additionally, the bromine moiety of PFOB also imparts radiopacity, enabling its detection using X-ray or CT imaging [21]. As an artificial oxygen carrier, the oxygen solubility in PFOB (44 mM) is 20-fold higher than that in water alone (2.2 mM) [22]. Thus, we hypothesize that PFOB/alginate-microencapsulated, allogeneic MSCs could survive *in vivo* without immunorejection and be detected by ^19^F MRI and CT longitudinally.

## 2. Methods

### 2.1. Microencapsulation of MSCs

All animal studies were approved by the Johns Hopkins University animal care and use committee to assure that all possible steps were taken to avoid animal suffering at each stage of the experiment. Rabbit MSCs were isolated from bone marrow of male New Zealand White (NZW) rabbits as previously described [15], and the culture was expanded *in vitro* for two passages prior to encapsulation or freezing.

MSC microencapsulation was performed using an electrostatic droplet generator as previously described [23–25]. Prior to encapsulation, PFOB was emulsified with an equal volume of lecithin (Sigma, St. Louis, MO) to make a homogenous stable solution. MSCs were then suspended at a concentration of 1-3 × 10^6^ cells/ml in a solution containing 12% (*w*/*v*) PFOB and 2% (*w*/*v*) high-viscosity guluronic acid (HVG) alginate (FMC BioPolymer, Philadelphia, PA). The PFOB/alginate mixture was passed through a 28G blunt tip needle at 0.05 ml/min into a solution containing 100 mM CaCl_2_. The microcapsules were collected and further incubated with 0.05% (*w*/*v*) poly-L-lysine and low-viscosity mannuronic acid alginate (FMC BioPolymer). Unlabeled microcapsules were made without PFOB. The viability of encapsulated MSCs was determined by a fluorescent live/dead staining using 2 *μ*M calcein AM (Trevigen, Gaithersburg, MD) and 5 mM propidium iodide (Invitrogen, Carlsbad, CA).

### 2.2. *In Vitro* Characterization of PFOB Microcapsules

The mechanical stability of the microcapsules was determined using swelling and osmotic pressure tests [26]. Aliquots of PFOB microcapsules (PFOBCaps) or unlabeled microcapsules were placed in 6 ml of 55 mM sodium citrate solution (Sigma) in a 6-well plate and incubated at 37°C for 2 hours. The swelling degree (*S*_w_) was assessed by measuring the changes in diameter of the microcapsules over time [27].

*S*_w_ is defined as follows:

(1) $$\begin{array}{c} S_{\text{w}}=\left[{\left(\frac{D_{t}}{D_{0}}\right)}^{3}-1\right]\times 100\%, \end{array}$$

where *D*_*t*_ and *D*_0_ were microcapsule diameters at time *t* and 0 (initial microcapsule diameter), respectively.

Osmotic pressure test was used to examine the mechanical stability of the microcapsules. To this end, PFOBCaps or unlabeled microcapsules were subjected to ultrapurified H_2_O incubation overnight at 37°C. The microcapsules were examined microscopically, and the percentage of intact microcapsules was recorded.

### 2.3. *In Vitro* Imaging of PFOBCaps

Two PFOBCap phantoms were designed using l.7% (*w*/*v*) agarose-filled, six-well culture plates to determine the detection limits of PFOBCaps using clinical flat-panel X-ray fluoroscopic system (Axiom Artis dFA, Forcheim, Germany) and 3T MRI (TIM Trio, Siemens, Erlangen, Germany). The first phantom contained PFOBCaps in groups of 2, 4, 6, 8, or 10 microcapsules per point, arranged in a 5-point dice pattern in each well. The second phantom contained PFOBCaps embedded with 5, 10, or 25 microcapsules per point creating the letters “JHU.”

C-arm CT images for both phantoms were acquired using the standard manufacturer presets (DynaCT Body, 8 s rotation; 240° scan angle; 0.5° increment; and 0.36 *μ*Gy dose per pulse, 70 kVp, and 48 cm field of view) and reconstructed using the vendor software. For the second phantom, ^19^F MRI was also acquired using a custom, four-channel phased array fluorine coil, which was tuned to the triplet peak of PFOB, and a 3D TrueFISP sequence (3.9 ms repetition time (TR); 2.0 ms echo time (TE); 1.5 × 1.5 × 2.0 mm^3^ voxel size; 1000 Hz/pixel bandwidth; 236 × 290 mm^2^ field of view; 2.0 mm slice thickness; and 15 min 43-second acquisition time).

### 2.4. *In Vivo* Imaging of PFOBCaps

To examine the *in vivo* detectability of PFOBCaps, we studied 31 female NZW rabbits divided into two cohorts. Cohort one included 15 normal rabbits whose right thigh received six intramuscular injections of PFOBCaps with MSCs (~0.02 ml/injection, ~1,000 PFOBCaps) and left thigh received six intramuscular injections of unencapsulated, fluorescently labeled (CellTracker Orange, Invitrogen, Carlsbad, CA) male rabbit MSCs. Cohort two included 16 PAD rabbits whose left superficial femoral (SFA) arteries were occluded by thrombogenic platinum coils (Vortex, Boston Scientific Cork Ltd., Cork, Ireland) using a nonsurgical, minimally invasive, endovascular method as previously described [15, 28]. These PAD rabbits received six injections of PFOBCaps with MSCs (0.4 ml/injection, ~20,000 PFOBCaps) in the left medial thigh three days after SFA occlusion to evaluate whether encapsulated MSCs could survive in the ischemic environment and whether other radiopaque materials presented in the body, such as bones and platinum coils, would affect the detectability of PFOBCaps.

All animals were sedated with intramuscular ketamine (40 mg/kg) and acepromazine (1 mg/kg) and induced with propofol to effect intravenously. Animals were intubated, and general anesthesia was maintained with 1%-2% isoflurane. Prior to imaging acquisition, animals were placed supine on a V-board to minimize movement between X-ray and MRI, and two rectangle PFOBCap reference markers were attached to the V-board posterior to the thighs of the rabbits.

For a sensitivity study (cohort one), X-ray angiograms, C-arm CTs, and ^19^F MR images were acquired in random order at 0, 1, 3, 7, and 14 days after PFOBCap injection using the same parameters as those described for phantoms. ^1^H MRIs (3D gradient echo, TR/TE = 15/3.7 ms; 1.0 × 0.9 × 1.5 mm^3^ voxel size; 320 Hz/pixel bandwidth; and 2 min acquisition) were acquired using the body coil for anatomical location followed by proton shimming. ^19^F MR images were acquired using the same 3D TrueFISP acquisition as that used in the phantom studies. A subset of animals was humanely euthanized at each time point after the imaging session for postmortem validation of the location of PFOBCaps.

For PAD rabbits (group two), X-ray angiograms, C-arm CT, and ^19^F MR images were obtained in random order at day 0, 7, and 14 after PFOBCap injections to monitor the delivery and distribution of encapsulated MSCs in the ischemic hind limb using parameters identical to above *in vivo* sensitivity studies. All animals were sacrificed 14 days after PFOBCap injections to assess the viability of encapsulated MSCs.

### 2.5. Postmortem Validation and Histopathological Analysis

To determine whether the signals detected on clinical C-arm CT images could be validated with PFOBCap injections, at each time point (days 0, 1, 3, 7, and 14), a minimum of two animals from cohort one were humanely euthanized after the imaging session, and their hind legs were perfusion fixed with 10% formalin. Each medial thigh was then dissected, sliced into 2.5 mm transverse sections, and digitally photographed. PFOBCap injection sites on postmortem gross sections were identified, and a portion of PFOBCaps were retrieved and placed in normal saline to examine the PFOBCap integrity and fibrotic tissue outgrowth. The injection sites on the digital images of each section were then manually contoured using ImageJ (v1.44). Injection site segmentation was performed using these contours to form a 3-dimensional volume of the injection sites, which, together with the digital image sections, were then coregistered with the corresponding C-arm CT volumetric image data using custom 3D visualization software (Dextroscope, Bracco AMT, Princeton, NJ). The six injection sites were used as landmark points to manually align the volumes to achieve optimal registration. The registration offset between paired centroids of the injection sites was then determined using vendor's software.

To assess MSC viability after transplantation, tissue blocks containing PFOB-encapsulated MSCs from both groups of animals were sectioned and terminal deoxynucleotidyl transferase dUTP nick end labeling (TUNEL) assay was performed on 20 *μ*m thick sections using an apoptosis kit (Millipore, Billerica, MA). The sections were counterstained with 4′,6-diamidino-2-phenylindole (DAPI). Fluorescence images were acquired using an inverted fluorescence microscope (Olympus XD51, Waltham, Massachusetts). To confirm the presence of male transplanted MSCs that were not encapsulated, suspected transplanted MSCs were first identified from hematoxylin and eosin- (H&E-) stained slides obtained around the injection sites, and then the cells were dissected from an adjacent slide using the laser capture microdissection system (Zeiss PALM, Thornwood, NY). Genomic DNA of the dissected samples was then isolated using QIAamp DNA FFPE Tissue kit (Qiagen), according to manufacturer's instructions. Rabbit MSC SRY and GAPDH DNAs were amplified by real-time PCR (Applied Biosystems) using KiCqstart SYBR green qPCR ready mix (Sigma). The primers used for SRY were 5′-3′-TTGGTTAGCACAAACCACCA (forward) and 5′-3′-AGCATTTTCCACTGGTGTCC (reverse). For GAPDH, the forward primer 5′-3′-GAATCCACTGGCGTCTTCAC and the reverse primer 5′-3′-CGTTGCTGACCATCTTGA-GAGA were used. The PCR products were analyzed on 2% agarose gel precast with ethidium bromide to confirm whether transplanted male MSCs were present. TUNEL staining was performed on an adjacent section after localizing transplanted male rabbit MSCs to assess whether transplanted cells remained alive.

### 2.6. Statistical Analysis

All continuous data are presented as mean ± standard deviation. The comparison of microcapsule stability and *in vitro* cell viability over time was performed using a cross-sectional, time series regression fitted with the random effects models by using the generalized least squares estimator (Stata 11, StataCorp LP, College Station, TX). A *P* value of <0.05 was considered statistically significant.

## 3. Results

### 3.1. MSC Encapsulation

**Figure 1:**
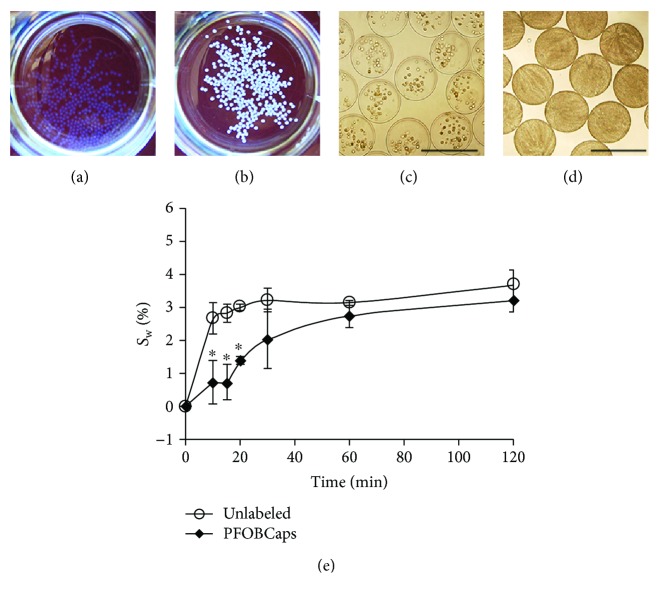
Macroscopic and microscopic images of rabbit MSC-containing microcapsules and their in vitro stability. (a) Unlabeled microcapsules appear transparent. (b) A digital image of PFOBCaps shows higher opacity than unlabeled microcapsules. (c) Light microscopic image of MSC-containing unlabeled microcapsules. (d) Microscopic image of MSC-containing PFOBCaps. (e) The swelling degree of PFOBCaps is significantly lower than that of unlabeled microcapsules in the first 20 minutes of incubation (*P* < 0.01). Scale bars represent 500 *μ*m.

Both unlabeled microcapsules (Figures 1(a) and 1(c)) and PFOBCaps (Figures 1(b) and 1(d)) were uniform in size (300 ± 16 *μ*m) and spherical with a smooth surface, which is ideal to minimize the activation of host immune response [29]. PFOBCaps appeared more opaque macroscopically (Figure 1(b)) and microscopically (Figure 1(d)) than unlabeled microcapsules due to the incorporation of the emulsified PFOB. Swelling tests showed significantly lower swelling degree for PFOBCaps than that for unlabeled microcapsules in the first 20 minutes (*P* < 0.01). The mean swelling degrees for both types of microcapsules were <3.7% during the two-hour study period (Figure 1(e)). During overnight osmotic pressure tests, both PFOBCaps and unlabeled microcapsules swelled significantly but no broken microcapsules were observed.

**Figure 2:**
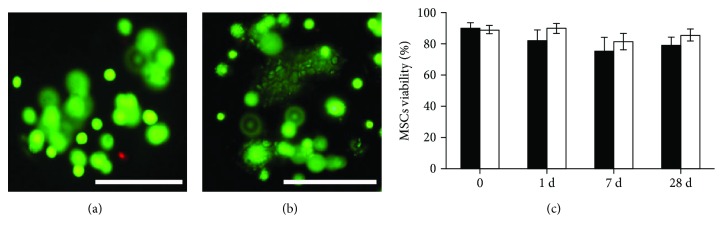
*In vitro* MSC viability assessment after encapsulation. (a, b) Photomicrographs of unlabeled microcapsules (a) and PFOBCaps (b) containing rabbit MSCs where live cells were stained green and dead cells were stained red. (c) Quantitative MSC viability in unlabeled (open bars) and PFOBCaps (black bars) at 0, 1, 7, and 28 days after encapsulation. The viability of encapsulated MSCs in both microcapsules did not differ significantly.

*In vitro* viability of encapsulated MSCs was high for both unlabeled microcapsule (Figure 2(a)) and PFOBCaps (Figure 2(b)) as indicated by the live/dead staining. Immediately after encapsulation, the viability of MSCs is 89.2 ± 2.7% in unlabeled microcapsules and 90.2 ± 3.1% in PFOBCaps. There was no significant further viability loss observed for both types of microcapsules up to 4 weeks (Figure 2(c)).

### 3.2. *In Vitro* Imaging of PFOBCaps

**Figure 3:**
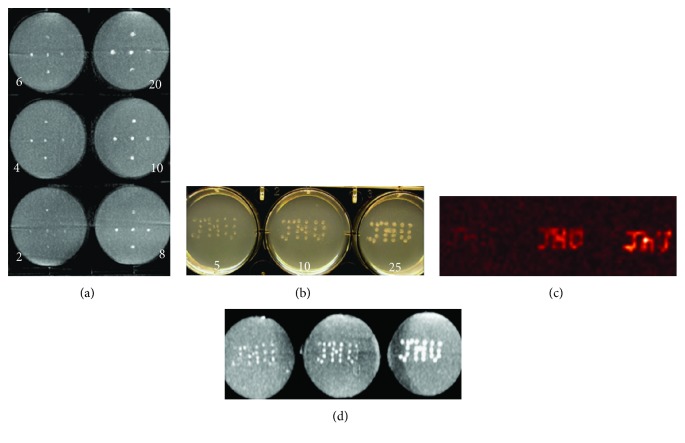
C-arm CT and ^19^F MR images of PFOBCap phantoms. (a) Maximum intensity projection (MIP) of C-arm CT image of a PFOBCap phantom containing 2, 4, 6, 8, 10, or 20 PFOBCaps/dot (number in white) arranged in five locations per well. As few as two PFOBCaps are readily seen in C-arm CT image. (b) A digital photograph of PFOBCap phantom with 5, 10, or 25 PFOBCaps/dot arranged in “JHU” pattern. (c) ^19^F MRI of PFOBCap phantom shows the detectability of as few as 10 PFOBCaps within less than 2 min acquisition time. (d) MIP of C-arm CT image of the same PFOBCap phantom as (a) showing the detection of all PFOBCaps.

*In vitro* phantom studies demonstrated that as few as two PFOBCaps could be visualized on C-arm CT images (Figure 3(a)). The sensitivity of ^19^F MR imaging to detect PFOBCaps in the phantom (Figure 3(b)) was high with as few as 10 PFOBCaps detected in <16 minutes of image acquisition time on a 3.0T clinical MR scanner (Figure 3(c)), which was further confirmed with C-arm CT images (Figure 3(d)).

### 3.3. *In Vivo* Visualization of PFOBCaps

**Figure 4:**
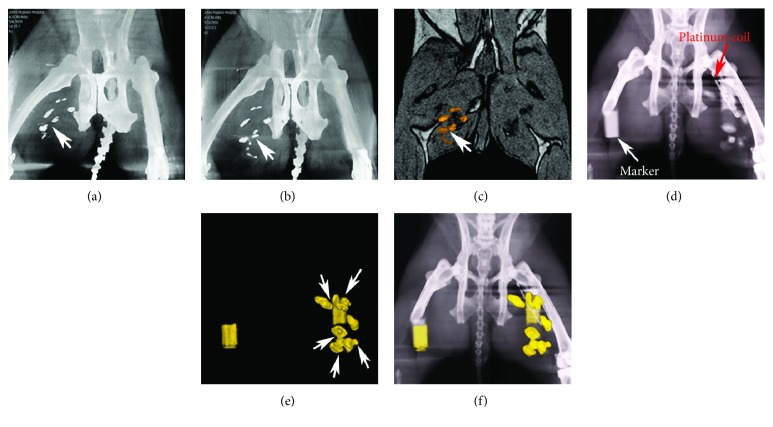
*In vivo* visualization of PFOBCaps in normal and PAD rabbits. (a) Multiplanar reformat of a C-arm CT in a normal rabbit demonstrates the detection of six PFOBCap injections in the right thigh (arrow) immediately after injection. (b) C-arm CT image of the same rabbit as (a) shows the persistence of PFOBCap injections two weeks after delivery. (c) *In vivo*^19^F MRI of PFOBCaps overlaid on anatomical ^1^H MRI shows the detection of PFOBCap injection sites two weeks after delivery. (d) C-arm CT image of a PAD rabbit shows additional radiopacities such as coil (dash line) beyond PFOBCaps. (e) ^19^F MRI of PFOBCaps in the same rabbit as (d) shows all six injections of PFOBCaps in the left thigh (arrows). (f) Fusion of (d) and (e) reveals one-to-one correspondence of PFOBCap injection sites.

Immediately after injection, 94.4% (85 out of 90) of attempted PFOBCap injection sites were detected in the right medial thigh of 15 normal rabbits by C-arm CT (Figure 4(a)). The radiopacities of detected PFOBCaps persisted for two weeks of postinjection (Figure 4(b)). ^19^F MRI demonstrated the sensitivity for detection of all PFOBCap injections shown on C-arm CT images and, when overlaid on anatomical ^1^H MR image, showed one-to-one correspondence of the location of PFOBCaps in the medial thigh of the rabbits (Figure 4(c)).

Although C-arm CT has high spatial resolution, in hind limb ischemia rabbits, the detection of PFOBCap injections was difficult due to the appearance of other radiodense materials, such as the platinum coils and bones (Figure 4(d)). Using ^19^F MRI, PFOBCap injections were clearly identified as “hot spots” without any background interference in PAD rabbits (Figure 4(e)). Fusion of C-arm CT images with^19^F MRI enabled unique identification of PFOBCap locations relative to other radiopaque structures (Figure 4(f)). Follow-up examinations demonstrated the persistence of the PFOBCaps on both C-arm CT and [19]F MRI two weeks after transplantation.

### 3.4. Postmortem Validation

**Figure 5:**
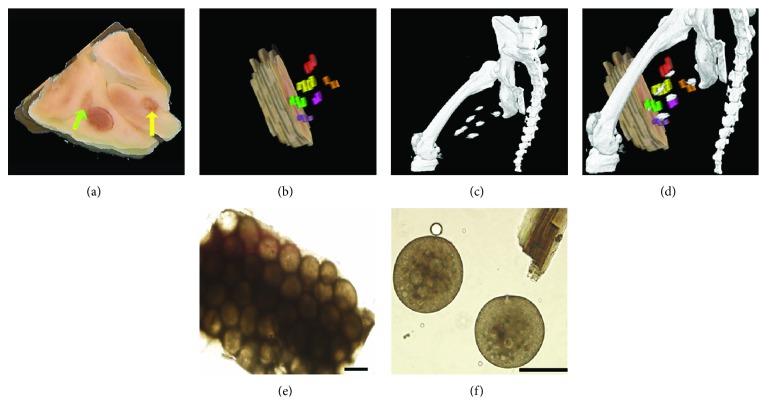
*Ex vivo* validation of PFOBCap injection sites. (a) Gross anatomical view of the rabbit medial thigh shows engrafted PFOBCaps (arrows) three days after implantation without hemorrhage or gross fibrosis. (b) Segmented injection sites with each injection show as a different color from postmortem tissue. (c) A C-arm CT image of the same rabbit demonstrates the detection of all six injections of PFOBCaps in the right thigh. (d) Fusion of C-arm CT image (c) with volume rendering of postmortem injections from (b) demonstrates high correlation of PFOBCap locations in the rabbit thigh. (e) Recovered PFOBCap injection site from rabbit medial thigh demonstrates that PFOBCaps remained intact two weeks after delivery. (f) Individual, intact PFOBCap recovered from tissue chunk two weeks after delivery. Scale bars represent 200 *μ*m.

Validation of the injection sites on C-arm CT images was performed in cohort one normal rabbits. A representative digital photograph of postmortem tissue slices acquired at three days after PFOBCap delivery demonstrated that there was no gross hemorrhage or fibrosis (Figures 5(a) and 5(b)). Out of 85 independent radiopaque regions on C-arm CT in 15 animals, 82 injection sites were identified postmortem. The three missing injection sites on postmortem tissue were attributed to tissue processing errors where the anatomical location was not retained, i.e., proximity of muscle to the femur. The location of PFOBCap injection sites, seen as radiopacities on C-arm CT (Figure 5(c)), demonstrated a high concordance with the postmortem locations as demonstrated in fused images (Figure 5d). The mean registration offset of PFOBCap injection sites between paired C-arm CT and postmortem was 2.83 ± 0.85 mm.

Two weeks after delivery, PFOBCaps could be readily extracted from the muscle bundles in the medial thigh when placed in saline (Figure 5(e)) and individual intact microcapsules could be clearly identified with minimal fibrotic tissue outgrowth around the microcapsules (Figure 5(f)).

### 3.5. Histopathology

**Figure 6:**
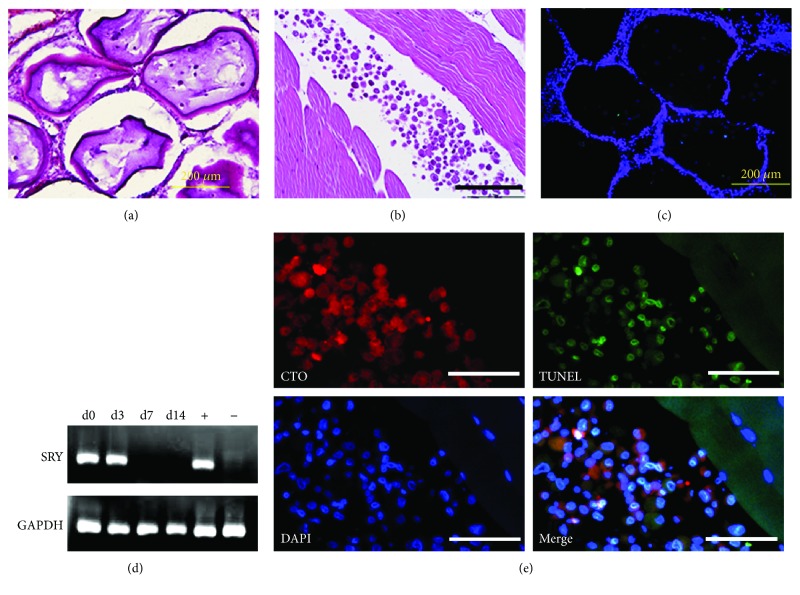
Histopathological analysis of MSC survival after transplantation. (a) Hematoxylin & eosin (H&E) staining of a rabbit receiving PFOBCaps with MSCsM shows absence of inflammatory cell infiltrate two weeks after delivery. (b) H&E staining of a rabbit receiving naked MSCs demonstrates no evidence of inflammatory infiltrates three days of postdelivery. (c) TUNEL staining reveals a few apoptotic MSCs (green, arrow) within PFOBCaps two weeks after transplantation. DAPI staining (blue) shows total cell nuclei. (d) qPCR analysis of Sry gene confirms the retention of male MSCs in rabbits up to three days of posttransplantation. Male rabbit MSCs serve as positive control (+) and female rabbit skeletal muscle cells serve as negative control (-). (e) TUNEL staining of CTO-labeled (orange) unencapsulated (or naked) MSCs reveals majority of cell death three days after delivery. Scale bars represent 200 *μ*m in (a–c) and 100 *μ*m in (e).

Histological analysis of H&E-stained sections showed no apparent foreign body reaction to allogeneic MSCs irrespective of whether the cells were encapsulated or not (Figures 6(a) and 6(b)). TUNEL staining (Figure 6(c)) of cryosections containing PFOBCaps with MSCs demonstrated that 56 ± 17% and 63 ± 10% of MSCs remained viable in the normal vs. ischemic PAD hind limbs, respectively, at two weeks after transplantation.

Quantitative PCR analysis confirmed the retention of naked male MSCs in rabbit tissue three days after transplantation (Figure 6(d)). However, TUNEL staining revealed that majority of naked MSCs were dead/apoptotic three days postdelivery (Figure 6(e)). No naked MSCs were detected at 7 and 14 days after transplantation.

## 4. Discussion

The multifunctional, perfluorinated microencapsulation formulation maintained allogeneic MSC viability even in the ischemic hind limbs and enabled immediate validation of delivery success and longitudinal tracking of encapsulated MSCs using clinical X-ray and MR systems. The dual-modality imaging visibility is of significant importance when considering long-term stem cell tracking. As most routine interventional procedures are X-ray- or ultrasound-guided, PFOB capsules, whose bromide moiety imparts radiopacity and whose perfluorocarbon moiety provides an echogenic ultrasound contrast agent [30], could be utilized for real-time monitoring of whether encapsulated MSCs are delivered successfully to targeted regions in PAD patients. Like radionuclide tracers, the introduction of PFOB into the alginate matrix provides positive “hot spot” ^19^F MR imaging capability with a high contrast-to-noise ratio and specificity due to the lack of endogenous fluorine signal in the body. Therefore, ^19^F MRI can be used for longitudinal tracking of PFOB-encapsulated MSCs while avoiding ionizing radiation to the patient or fragile stem cells.

Currently, most cellular clinical trials for PAD patients employ autologous stem/progenitor cells isolated from bone marrow or peripheral blood [31–33]. However, a bulk of evidence suggests that patients with coronary or peripheral atherosclerosis often suffer from a reduction and dysfunction of stem/progenitor cells [8, 34, 35], especially in the presence of diabetes mellitus, which in turn may limit the success of autologous cell therapy. From this perspective, allogeneic cells from healthy donors may provide a better cellular therapeutic for acute treatment. While allogeneic cells may raise concerns of immunorejection, methods for physically isolating the cells from the host immune system have been developed, of which islet microencapsulation with permeability tunable alginate hydrogel has moved into clinical trials for type I diabetes treatment [17, 36, 37]. Cellular microencapsulation essentially isolates the MSCs immunologically. The pores within the microcapsules are large enough to permit the free diffusion of nutrients and cytokines, but small enough to prevent antibody or immune cell entry [38]. While such selective permeability may block the effusion of certain beneficial large size substances such as exosomes, the enhanced angiogenesis and arteriogenesis as demonstrated in the early study suggest a paracrine mechanism presumably via small molecule angiogenic or proangiogenic factors secreted by the encapsulated MSCs [15].

Alginate is a natural linear polysaccharide consisted of similar and strictly alternating blocks of *β*-(1 → 4)-linked D-mannuronic acid (M) and *α*-(1 → 4)-linked L-guluronic acid (G) residues. In the presence of low concentration of divalent cations, such as Ca^2+^and Ba^2+^, alginate forms a soft gel readily through stereocomplex formation between G residues of the polymer [39]. Previous studies have demonstrated the biocompatibility profile of alginate in several biomedical applications such as tissue-engineered cardiac graft [40], cell delivery carriers [12, 13], and wound dressing [41]. However, clinical studies of alginate-encapsulated islet transplantation have resulted in strong fibrotic overgrowth around the microcapsules, suggesting poor biocompatibility of alginate microcapsules. This is in contrast to the findings in the present study where high cell survival rate and the lack of foreign body reaction were noted during the two-week study period. The possible explanations may lie with the high purity of the alginate we used [16] and the immune regulatory properties of MSCs encapsulated [42].

Previous clinical and preclinical studies on tracking stem cell therapy have been mainly relied on direct cell labeling with imaging contrast agents, such as superparamagnetic iron oxide (SPIO) and ^19^F emulsions [20, 43–45]. However, concerns about the effect of the labels on stem cell viability and differentiation capacity remain. It has been recognized that direct SPIO labeling inhibits MSC chondrogenesis in a dose-dependent manner [43] and leads to a decrease in MSC migration and colony formation [46]. By converting direct cell labeling into microcapsule labeling, high payload of contrast agents, which would normally be cytotoxic, can now be used to enable cell tracking with high sensitivity [47]. We have previously demonstrated the feasibility of applying barium sulfate-impregnated microcapsules for X-ray monitoring of MSC delivery in PAD rabbits [15] and immunocompetent swine [23]. However, the potential toxicity related to heavy metal raises concerns for clinical translation. In addition, physiological oxygen levels within microcapsules are often significantly lower than those of *in vitro* culturing systems, which may negatively affect cellular metabolic activities [48], especially in disease where an ischemic environment is anticipated. In this study, PFOB was selected as an imaging contrast as it has been clinically studied in patients as a blood substitute and has been demonstrated to improve the viability of immobilized HepG2 cells within hydrogel scaffolds *in vitro* [49]. The enhanced *in vivo* MSC viability as compared to naked MSCs in this study is likely attributed, in part, to the immunoprotection of microcapsules, the effectiveness of PFOB on elevating oxygen availability to encapsulated MSCs, or these combined effects.

Transplanted naked MSCs even with fluorophore labels are often difficult to identify in relatively large tissue. The sex mismatched transplantation as designed in this study enabled the use of male specific gene, Sry, to validate the presence of transplanted male MSCs in female rabbit tissue. In our study, qPCR results showed no detection of transplanted MSCs beyond three days after transplantation (Figure 6(d)), which might be attributed to the significant early cell death as demonstrated by TUNEL staining (Figure 6(e)). If naked transplanted MSCs only survive for <3 days in normal muscle, then the dosing schedule will be critical relative to encapsulated MSCs which demonstrated a 14-day survival even in the ischemic rabbit model.

Although ^19^F is not as abundant as ^1^H in animals or humans, the detection sensitivity we observed on a 3.0T clinical MR scanner was sufficient to detect small numbers of injected PFOBCaps. In fact, the number of fluorine atoms in one voxel of ^19^F MRI of PFOBCaps was approximately on the order of 10 [19], which is ten times higher than direct ^19^F-labeled stem cells and stromal vascular fraction cells [20, 45]. Moreover, by superimposing ^19^F MR image on anatomical ^1^H MR image, the precise location of microencapsulated MSCs could be determined. Therefore, PFOB impregnation of microcapsules could be advantageous to perform longitudinal follow-up examinations with ^19^F MRI without extended ionizing radiation exposure.

Successfully injected PFOBCaps were all visualized on C-arm CT or ^19^F MRI images. Approximately 6% attempted injections were not detected, which is attributed to failed PFOBCap delivery rather than detection issues. Since there was tissue deformation during fixation, a small registration offset of the injection sites between paired C-arm CT and postmortem images was observed but probably of insignificant importance in targeting accuracy. Because PFOBCaps were made of pharmaceutical grade, ultrapurified alginate, and perfluorocarbon (i.e., PFOB), and the imaging data were obtained in large animals using clinical scanners, the present work could form the basis for direct translation of PFOBCaps into clinical therapeutic angiogenesis treatment for PAD or other ischemic arterial diseases, such as myocardial infarction.

As the current *in vivo* study lasted only 14 days, the long-term biocompatibility and stability of PFOBCaps *in vivo* will require additional studies. Fortunately, the dual-imaging detectability enables noninvasive monitoring of the integrity of PFOBCaps. Additionally, future studies involving combinational immunohistochemistry, PCR analysis, and TIMI frame count to determine whether the high survival rate of encapsulated MSCs correlates with the therapeutic efficacy in PAD animal model will be warranted prior to clinical applications.

## 5. Conclusions

The current study presents the first application of dual-modality-visible, perflorinated microcapsule for precise monitoring of allogeneic MSC delivery and tracking the distribution of encapsulated MSCs in a large animal model using clinical imaging systems. PFOBCaps maintain high survival of allogeneic MSCs even in the ischemic tissue, whereas nonencapsulated allogeneic MSCs failed to survive longer than 3 days of postadministration.

## Data Availability

All data used to support the findings of this study are available from the corresponding author upon request.

## References

[1] Benjamin E. J., Virani S. S., Callaway C. W. (2018). Heart disease and stroke statistics-2018 update: a report from the American Heart Association. *Circulation*.

[2] Hooi J. D., Kester A. D., Stoffers H. E. J. H., Rinkens P. E. L. M., Knottnerus J. A., van Ree J. W. (2004). Asymptomatic peripheral arterial occlusive disease predicted cardiovascular morbidity and mortality in a 7-year follow-up study. *Journal of Clinical Epidemiology*.

[3] Kalka C., Baumgartner I. (2008). Gene and stem cell therapy in peripheral arterial occlusive disease. *Vascular Medicine*.

[4] Tournois C., Pignon B., Sevestre M. A. (2017). Cell therapy in critical limb ischemia: a comprehensive analysis of two cell therapy products. *Cytotherapy*.

[5] Wang Z. X., Li D., Cao J. X. (2014). Efficacy of autologous bone marrow mononuclear cell therapy in patients with peripheral arterial disease. *Journal of Atherosclerosis and Thrombosis*.

[6] Tateishi-Yuyama E., Matsubara H., Murohara T. (2002). Therapeutic angiogenesis for patients with limb ischaemia by autologous transplantation of bone-marrow cells: a pilot study and a randomised controlled trial. *Lancet*.

[7] Fadini G. P., Miorin M., Facco M. (2005). Circulating endothelial progenitor cells are reduced in peripheral vascular complications of type 2 diabetes mellitus. *Journal of the American College of Cardiology*.

[8] Heeschen C., Lehmann R., Honold J. (2004). Profoundly reduced neovascularization capacity of bone marrow mononuclear cells derived from patients with chronic ischemic heart disease. *Circulation*.

[9] Zhang M., Methot D., Poppa V., Fujio Y., Walsh K., Murry C. E. (2001). Cardiomyocyte grafting for cardiac repair: graft cell death and anti-death strategies. *Journal of Molecular and Cellular Cardiology*.

[10] van der Bogt K. E., Sheikh A. Y., Schrepfer S. (2008). Comparison of different adult stem cell types for treatment of myocardial ischemia. *Circulation*.

[11] Qiao H., Surti S., Choi S. R. (2009). Death and proliferation time course of stem cells transplanted in the myocardium. *Molecular Imaging and Biology*.

[12] Lim F., Sun A. M. (1980). Microencapsulated islets as bioartificial endocrine pancreas. *Science*.

[13] Barnett B. P., Arepally A., Karmarkar P. V. (2007). Magnetic resonance-guided, real-time targeted delivery and imaging of magnetocapsules immunoprotecting pancreatic islet cells. *Nature Medicine*.

[14] Abalovich A. G., Bacqué M. C., Grana D., Grana D., Milei J. (2009). Pig pancreatic islet transplantation into spontaneously diabetic dogs. *Transplantation Proceedings*.

[15] Kedziorek D. A., Hofmann L. V., Fu Y. (2012). X-ray-visible microcapsules containing mesenchymal stem cells improve hind limb perfusion in a rabbit model of peripheral arterial disease. *Stem Cells*.

[16] Mallett A. G., Korbutt G. S. (2009). Alginate modification improves long-term survival and function of transplanted encapsulated islets. *Tissue engineering Part A*.

[17] Tuch B. E., Keogh G. W., Williams L. J. (2009). Safety and viability of microencapsulated human islets transplanted into diabetic humans. *Diabetes Care*.

[18] de Groot M., Schuurs T. A., van Schilfgaarde R. (2004). Causes of limited survival of microencapsulated pancreatic islet grafts. *The Journal of Surgical Research*.

[19] Barnett B. P., Ruiz-Cabello J., Hota P. (2011). Use of perfluorocarbon nanoparticles for non-invasive multimodal cell tracking of human pancreatic islets. *Contrast Media & Molecular Imaging*.

[20] Rose L. C., Kadayakkara D. K., Wang G. (2015). Fluorine-19 labeling of stromal vascular fraction cells for clinical imaging applications. *Stem Cells Translational Medicine*.

[21] Wang G., Fu Y., Shea S. M., Hegde S. S., Kraitchman D. L. (2018). Quantitative CT and ^19^F-MRI tracking of perfluorinated encapsulated mesenchymal stem cells to assess graft immunorejection. *Magnetic Resonance Materials in Physics, Biology and Medicine*.

[22] Khattak S. F., Chin K. S., Bhatia S. R., Roberts S. C. (2007). Enhancing oxygen tension and cellular function in alginate cell encapsulation devices through the use of perfluorocarbons. *Biotechnology and Bioengineering*.

[23] Fu Y., Azene N., Ehtiati T. (2014). Fused X-ray and MR imaging guidance of intrapericardial delivery of microencapsulated human mesenchymal stem cells in immunocompetent swine. *Radiology*.

[24] Kedziorek D. A., Solaiyappan M., Walczak P. (2013). Using C-arm X-ray imaging to guide local reporter probe delivery for tracking stem cell engraftment. *Theranostics.*.

[25] Barnett B. P., Arepally A., Stuber M., Arifin D. R., Kraitchman D. L., Bulte J. W. M. (2011). Synthesis of magnetic resonance-, X-ray- and ultrasound-visible alginate microcapsules for immunoisolation and noninvasive imaging of cellular therapeutics. *Nature Protocols*.

[26] Van Raamsdonk J. M., Chang P. L. (2001). Osmotic pressure test: a simple, quantitative method to assess the mechanical stability of alginate microcapsules. *Journal of Biomedical Materials Research*.

[27] Liu X., Xue W., Liu Q. (2004). Swelling behaviour of alginate–chitosan microcapsules prepared by external gelation or internal gelation technology. *Carbohydrate Polymers*.

[28] Liddell R. P., Patel T. H., Weiss C. R. (2005). Endovascular model of rabbit hindlimb ischemia: a platform to evaluate therapeutic angiogenesis. *Journal of Vascular and Interventional Radiology*.

[29] Dufrane D., Goebbels R. M., Saliez A., Guiot Y., Gianello P. (2006). Six-month survival of microencapsulated pig islets and alginate biocompatibility in primates: proof of concept. *Transplantation*.

[30] Mattrey R. F. (1989). Perfluorooctylbromide: a new contrast agent for CT, sonography, and MR imaging. *AJR. American Journal of Roentgenology*.

[31] Perin E. C., Murphy M. P., March K. L. (2017). Evaluation of cell therapy on exercise performance and limb perfusion in peripheral artery disease: the CCTRN PACE trial (patients with intermittent claudication injected with ALDH bright cells). *Circulation*.

[32] Peeters Weem S. M., Teraa M., de Borst G. J., Verhaar M. C., Moll F. L. (2015). Bone marrow derived cell therapy in critical limb ischemia: a meta-analysis of randomized placebo controlled trials. *European Journal of Vascular and Endovascular Surgery*.

[33] Liotta F., Annunziato F., Castellani S. (2018). Therapeutic efficacy of autologous non-mobilized enriched circulating endothelial progenitors in patients with critical limb ischemia- the SCELTA trial.. *Circulation Journal*.

[34] Prochazka V., Gumulec J., Jalůvka F. (2010). Cell therapy, a new standard in management of chronic critical limb ischemia and foot ulcer. *Cell Transplantation*.

[35] Capla J. M., Grogan R. H., Callaghan M. J. (2007). Diabetes impairs endothelial progenitor cell-mediated blood vessel formation in response to hypoxia. *Plastic and reconstructive surgery.*.

[36] Soon-Shiong P., Heintz R. E., Merideth N. (1994). Insulin independence in a type 1 diabetic patient after encapsulated islet transplantation. *The Lancet*.

[37] Calafiore R., Basta G., Luca G. (2006). Microencapsulated pancreatic islet allografts into nonimmunosuppressed patients with type 1 diabetes: first two cases. *Diabetes Care*.

[38] Barnett B. P., Kraitchman D. L., Lauzon C. (2006). Radiopaque alginate microcapsules for X-ray visualization and immunoprotection of cellular therapeutics. *Molecular Pharmaceutics*.

[39] Poojari R., Srivastava R. (2013). Composite alginate microspheres as the next-generation egg-box carriers for biomacromolecules delivery. *Expert Opinion on Drug Delivery*.

[40] Perets A., Baruch Y., Weisbuch F., Shoshany G., Neufeld G., Cohen S. (2003). Enhancing the vascularization of three-dimensional porous alginate scaffolds by incorporating controlled release basic fibroblast growth factor microspheres. *Journal of Biomedical Materials Research. Part A*.

[41] Thomas S. (2000). Alginate dressings in surgery and wound management--part 1. *Journal of Wound Care*.

[42] Davis N. E., Hamilton D., Fontaine M. J. (2012). Harnessing the immunomodulatory and tissue repair properties of mesenchymal stem cells to restore *β* cell function. *Current diabetes reports*.

[43] Kostura L., Kraitchman D. L., Mackay A. M., Pittenger M. F., Bulte J. W. M. (2004). Feridex labeling of mesenchymal stem cells inhibits chondrogenesis but not adipogenesis or osteogenesis. *NMR in Biomedicine*.

[44] Goodfellow F., Simchick G. A., Mortensen L. J., Stice S. L., Zhao Q. (2016). Tracking and quantification of magnetically labeled stem cells using magnetic resonance imaging. *Advanced Functional Materials*.

[45] Boehm-Sturm P., Mengler L., Wecker S., Hoehn M., Kallur T. (2011). In vivo tracking of human neural stem cells with 19F magnetic resonance imaging. *PLoS One*.

[46] Schafer R., Kehlbach R., Müller M. (2009). Labeling of human mesenchymal stromal cells with superparamagnetic iron oxide leads to a decrease in migration capacity and colony formation ability. *Cytotherapy*.

[47] Arifin D. R., Kedziorek D. A., Fu Y. (2013). Microencapsulated cell tracking. *NMR in Biomedicine*.

[48] Mukundan N. E., Flanders P. C., Constantinidis I., Papas K. K., Sambanis A. (1995). Oxygen consumption rates of free and alginate-entrapped beta TC3 mouse insulinoma cells. *Biochemical and Biophysical Research Communications*.

[49] Chin K., Khattak S. F., Bhatia S. R., Roberts S. C. (2008). Hydrogel-perfluorocarbon composite scaffold promotes oxygen transport to immobilized cells. *Biotechnology Progress*.

